# Computational study of the balloon dilation steps on transcatheter aortic valve replacement

**DOI:** 10.3389/fbioe.2023.1333138

**Published:** 2023-12-20

**Authors:** Jianming Li, Zhuangyuan Meng, Wentao Yan, Wenshuo Wang, Lai Wei, Shengzhang Wang

**Affiliations:** ^1^ Department of Aeronautics and Astronautics, Institute of Biomechanics, Fudan University, Shanghai, China; ^2^ Shanghai Inspection and Research Institute for Medical Devices, Shanghai, China; ^3^ Department of Cardiac Surgery, Zhongshan Hospital Affiliated to Fudan University, Shanghai, China; ^4^ Academy for Engineering and Technology, Institute of Biomedical Engineering Technology, Fudan University, Shanghai, China; ^5^ Zhuhai Fudan Innovation Institute, Zhuhai, China

**Keywords:** transcatheter aortic valve replacement, numerical simulation, self-expandable valve, post-dilation, pre-dilation

## Abstract

Balloon dilation is a commonly used assistant method in transcatheter aortic valve replacement (TAVR) and plays an important role during valve implantation procedure. The balloon dilation steps need to be fully considered in TAVR numerical simulations. This study aims to establish a TAVR simulation procedure with two different balloon dilation steps to analyze the impact of balloon dilation on the results of TAVR implantation. Two cases of aortic stenosis were constructed based on medical images. An implantation simulation procedure with self-expandable valve was established, and multiple models including different simulation steps such as balloon pre-dilation and balloon post-dilation were constructed to compare the different effects on vascular stress, stent morphology and paravalvular leakage. Results show that balloon pre-dilation of TAVR makes less impact on post-operative outcomes, while post-dilation can effectively improve the implantation morphology of the stent, which is beneficial to the function and durability of the valve. It can effectively improve the adhesion of the stent and reduce the paravalvular leakage volume more than 30% after implantation. However, balloon post-dilation may also lead to about 20% or more increased stress on the aorta and increase the risk of damage. The balloon dilation makes an important impact on the TAVR outcomes. Balloon dilation needs to be fully considered during pre-operative analysis to obtain a better clinical result.

## 1 Introduction

Aortic stenosis is a common heart disease caused by aortic valve calcification. Aortic stenosis causes reduction of cardiac output and may be accompanied by aortic regurgitation (AR), resulting in systemic insufficiency of blood supply, which may lead to cardiac hypertrophy and severe failure in long term (Pibarot et al., 2019; Spitzer et al., 2019). Transcatheter aortic valve replacement (TAVR) is a minimally invasive treatment technique which delivers the compressible artificial valve through a microcatheter to complete the replacement. It has been widely used in the treatment of high-risk patients. However, due to factors such as non-intuitive surgery and complex anatomy, post-operative complications are the main problems. Atrioventricular conduction may be disturbed by the contact stimulation from prosthetic stent, which needs to be regulated by implantation of pacemaker. The existence of post-operative paravalvular leak is related to higher late mortality, cardiac death and secondary hospitalization (Maisano et al., 2015). In addition, paravalvular leak is also associated with the formation of thrombosis, thus increasing the risk of post-operative stroke (Bianchi et al., 2019).

Clinically, due to the severity of valve stenosis, the valve stent cannot pass smoothly. Therefore, the operator will use balloon dilation to fully open the original valve before valve implantation, which is called balloon pre-dilation. After the valve is released, a balloon is used again for secondary dilation, which is called post-dilation, allowing the valve stent to fully expand to achieve support. These two steps usually play an important role in TAVR procedure (McInerney et al., 2021).

Computational modelling and simulation have been widely used to study TAVR, such as the relationship between calcification distribution and TAVR results and the influence of stent positioning (Auricchio et al., 2014; Morganti et al., 2016; Sturla et al., 2016; Vy et al., 2016; Luraghi et al., 2020). In addition, numerical simulation can be used to evaluate the risk of different operation strategy and predict post-operative outcomes. Morganti et al. (Morganti et al., 2014) established the implantation process of balloon-expandable valve based on two patient-specific models, and then established the simulation model of self-expandable valve to study the influence of valve positioning on the post-operative outcomes (Morganti et al., 2016). Using a combination method of structural simulation and computational fluid dynamics, Bianchi et al. (Bianchi et al., 2019) pointed out the effective reduction of post-operative paravalvular leak with over-dilation for balloon-expandable valve.

However, in many TAVR simulation studys, the balloon dilation steps were not mentioned in detail or considered in TAVR simulation, which typically included two main steps: stent crimping and releasing. The effect of balloon dilation on simulation result is not clear so far. In this study, two patient-specific aortic models were established, and a complete transcatheter self-expandable valve implantation process would be constructed in the model to verify the effectiveness of the balloon dilation step and analyze the impact of balloon dilation on the post-operative results.

## 2 Methods

### 2.1 Aortic root and TAVR models

**FIGURE 1:**
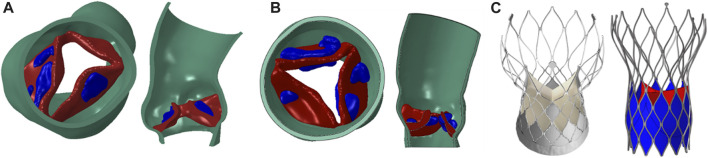
Aortic models and prosthetic valve models. **(A)** aortic model for CASE 1 (green part for aortic wall, red part for native valve, blue part for calcification); **(B)** aortic model for CASE 2; **(C)** TaurusOne self-expandable valve and numerical model.

In this study, CTA image data of two patients diagnosed with severe aortic stenosis were retrospectively analyzed and chosen for model reconstruction. Patient-specific aortic models were reconstructed in MIMICS 19.0 (Materialise, Belgium) from the left ventricular outflow tract to the ascending aorta, including the aortic wall, native valve and calcification. The models were repaired and smoothed in Geomagic Studio 2013 (Geomagic Inc. United States) to generate a standard geometry format, and finally meshed in Hypermesh 2019 (Altair, United States), as shown in Figures 1A,B. The native valve and calcification were meshed with tetrahedral elements. The aortic wall was meshed with triangles on inner surface and then offset outward to generate a solid mesh with total thickness of 1.5 mm. The total number of elements of each model was about 250,000, which satisfied the mesh independence requirement (Li et al., 2022). In the process of TAVR simulation, aortic model can be assumed as linear elastic material (Bailey et al., 2016) to reduce the computational cost, and detailed material parameters were listed in Table 1. The circumference of native annulus was 71.7 mm and 73.7 mm for two cases, respectively, corresponding to a diameter of 22.8 mm and 23.8 mm. Therefore, according to the clinical recommendations, a 26 mm size self-expandable valve was chosen for implantation simulation for both two cases.

**TABLE 1:** Material parameters for aortic model.

Component	Elastic modulus (MPa)	Poisson’s ratio
Aortic wall	2	0.45
Leaflets	3.3	0.45
Calcification	12.6	0.3

Based on the commercial transcatheter heart valve products TaurusOne (Peijia Medical Inc. China), the self-expandable valve model (TO) used in this study was constructed, as shown in Figure 1C. The self-expandable stent was made of hyperelastic nitinol alloy material, which involves a phase transformation between Austenite and Martensite during loading and unloading process. The properties of nitinol were defined in Abaqus 2019 (Dassault System, France) by VUMAT. Parameters for these two stents are listed in Table 2.

**TABLE 2:** Material parameters for self-expandable stent.

Parameter	Description	Value
$E_{A}$	Austenite elastic modulus	45,000 MPa
$\nu_{A}$	Austenite Poisson’s ratio	0.33
$E_{M}$	Martensite elastic modulus	30,000 MPa
$\nu_{M}$	Martensite Poisson’s ratio	0.33
$\varepsilon^{L}$	Transformation strain	0.032
$\sigma_{L}^{s}$	Start of transformation loading	250 MPa
$\sigma_{L}^{E}$	End of transformation loading	550 MPa
$\sigma_{U}^{s}$	Start of transformation unloading	60 MPa
$\sigma_{U}^{E}$	End of transformation unloading	20 MPa
ρ	Material density	4,500 kg/m^3^

### 2.2 TAVR implantation procedure

**FIGURE 2:**
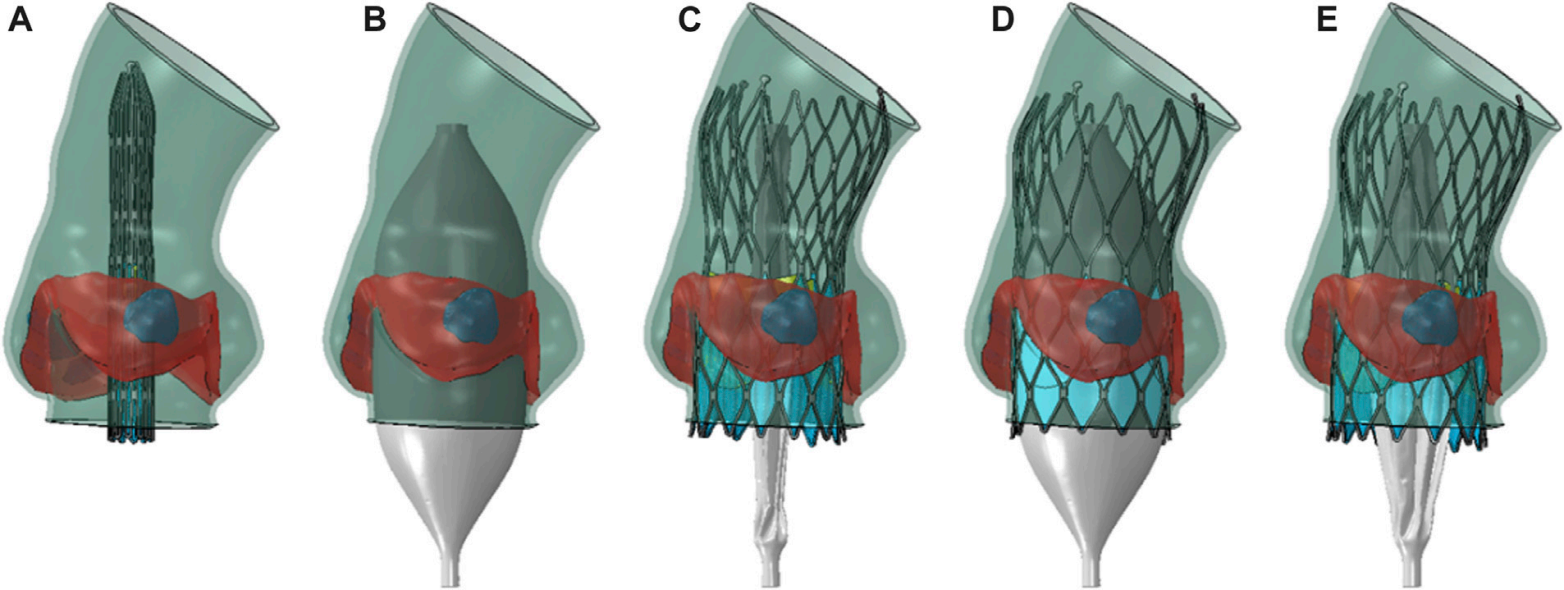
TAVR implantation process. **(A)** valve crimping; **(B)** balloon pre-dilation; **(C)** stent releasing; **(D)** balloon post-dilation; **(E)** balloon retraction.

Nitinol can autonomously return to its original shape after the constraints are removed, and the valve implantation process is generally completed with crimping and releasing steps (Morganti et al., 2016; Nappi et al., 2021). In this study, the complete TAVR process in clinical operation was restored, and the steps of balloon pre-dilation and post-dilation were added to simulate a more realistic self-expandable valve implantation process. The complete self-expandable valve implantation process established was shown in Figure 2.

1. Valve crimping: A rigid cylindrical tube and the prosthetic valve were assembled concentrically and adjusted to the appropriate implantation height. A radially inward displacement boundary condition was applied to the cylindrical tube surface, causing it to shrink radially to a diameter of 7 mm. The general contact between the stent, the skirt and the rigid cylindrical surface was set. The radius of the valve was reduced under the driving of the rigid cylinder to obtain the crimped state.
2. Balloon pre-dilation: A 23 mm balloon was inflated using the method of fluid cavity, which increases the volume of liquid inside the balloon to reach the inflation state. The contact happened among the balloon, the native valve and the aortic wall, causing the native valve to open. In order to improve the calculation efficiency, the calculation of this step was performed simultaneously with the stent crimping step, by setting no contact between the TAVR valve and the aortic model to make them two independent processes.
3. Stent releasing: In this step, the volume of liquid inside the balloon was reduced to deflate the pre-dilation balloon. The balloon gradually shrunk under the action of external pressure and elastic recovery, and the native valve and aortic wall recovered. In the meanwhile, a radial outward displacement condition was applied to the rigid cylindrical tube to increase its diameter. Due to the superelasticity of the material, the self-expandable stent gradually recovered its shape and came into contact with the native valve and aortic wall. Finally, the constraint of the rigid cylindrical tube on the prosthetic valve was released, and the valve interacted with the aorta to reach a stable state.
4. Balloon post-dilation: Second balloon dilation was performed after the valve released completely. During this process, the balloon surface interacted with the self-expandable valve and the aorta and lead to deformation. Inter-contacts and self-contacts of all components in the model were taken into account.
5. Balloon retraction: The post-dilation balloon gradually retracted. Due to the elimination of internal support by the balloon, the self-expandable valve slight contracted under the elastic recovery force of the aortic root and native valve, and eventually the whole model reached a final balance.

In order to study the impact of balloon dilation on the implantation outcomes with self-expandable valve, adjustments were made in the simulation procedure to establish four compared models, without balloon dilation (TO), only considering balloon pre-dilation (TO-pre), only considering balloon post-dilation (TO-post), both considering pre-dilation and post-dilation (TO-prepost).

### 2.3 Calculation of stent deformation and paravalvular leak

**FIGURE 3:**
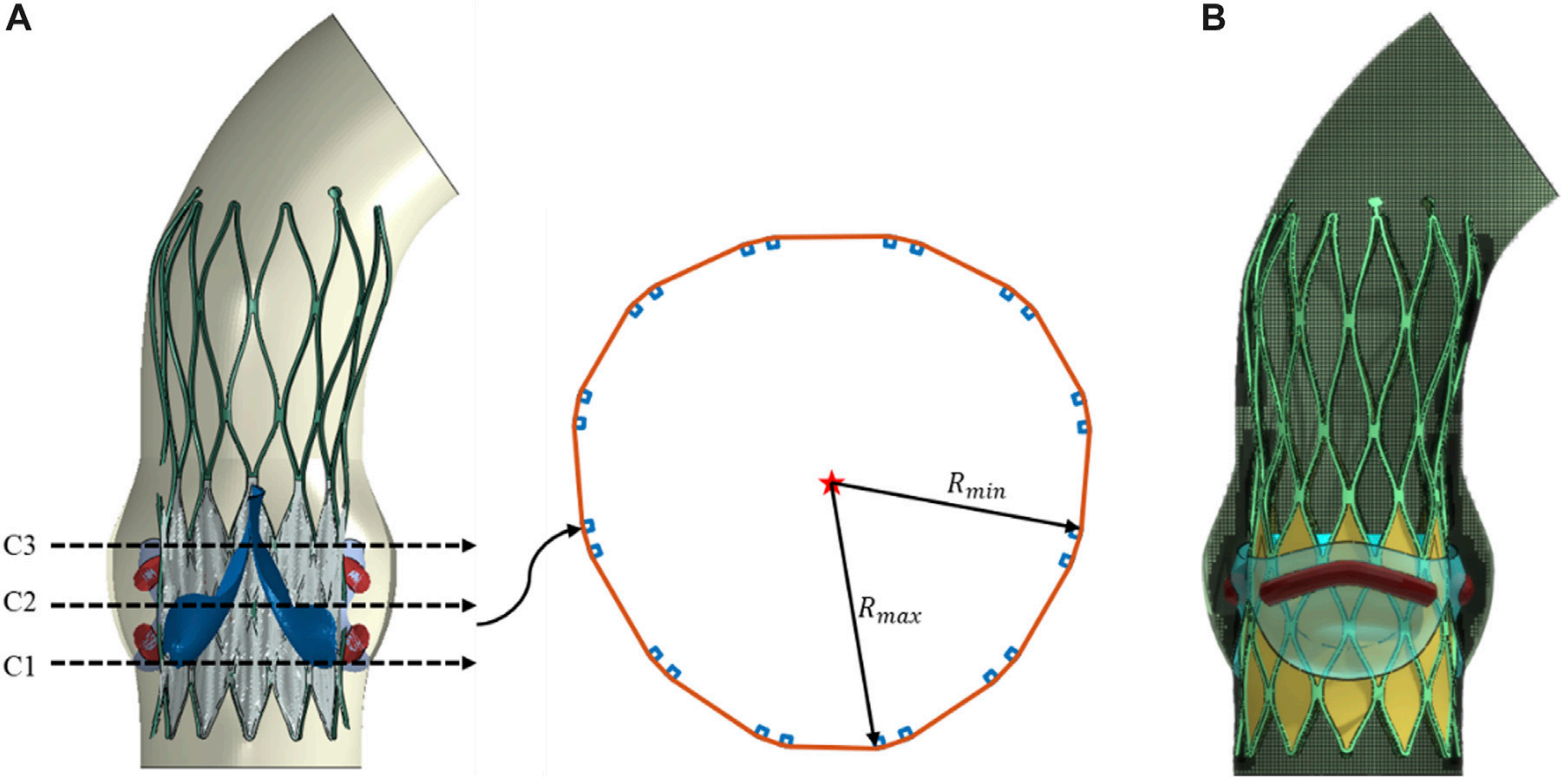
TAVR stent deformation and paravalvular leak calculation diagram. **(A)** eccentricity of stent cross-section; **(B)** LBM calculation lattice.

Three valve frame sections with from the area of the sewn prosthetic valve were extracted for deformation analysis after valve implantation (Nappi et al., 2021). The cross-sectional eccentricity of the stent was used to evaluate the degree of deformation of the stent, which was calculated as R_max_/R_min_, represented the ratio of the maximum to minimum distance from the point on the cross-section to the geometric center, as shown in Figure 3 (a).

Computational fluid dynamics (CFD) was used for post-operative paravalvular leak analysis. Due to the tiny penetrations or gaps in the models after valve implantation, it was difficult to construct an effective and stable computational mesh using traditional CFD methods, such as finite volume method. Therefore, the Lattice Boltzmann Method (LBM) was employed to calculate the regurgitation caused by paravalvular leak. LBM is a meshless method, which is easy to match with complex geometric boundaries. The calculation was completed in the software XFlow 2019 (Dassault System, France).

Through mesh convergence analysis using regurgitation flow as the inspection index, the global lattice size was finally determined to be 0.4 mm, with local refinement to 0.1 mm on the stent wall and native valve. Paravalvular leak primarily occurs during diastole, when blood flows to the ventricle side from the aorta. The calculation was considered as steady flow, with average diastolic aortic pressure of 13,500 Pa applying for inlet condition in ascending aorta side and diastolic left ventricular pressure of 500 Pa applying for outlet in ventricle side. Blood was assumed as Newtonian fluid with density of 1,050 kg/m3 and kinematic viscosity of 0.0035 Pa∙s. In order to calculated the final regurgitation degree in clinical usage, thecardiaccycle was assumed as 0.8 s and the diastolic period accounted for about 65%.

## 3 Results

### 3.1 Aortic stress distribution

**FIGURE 4:**
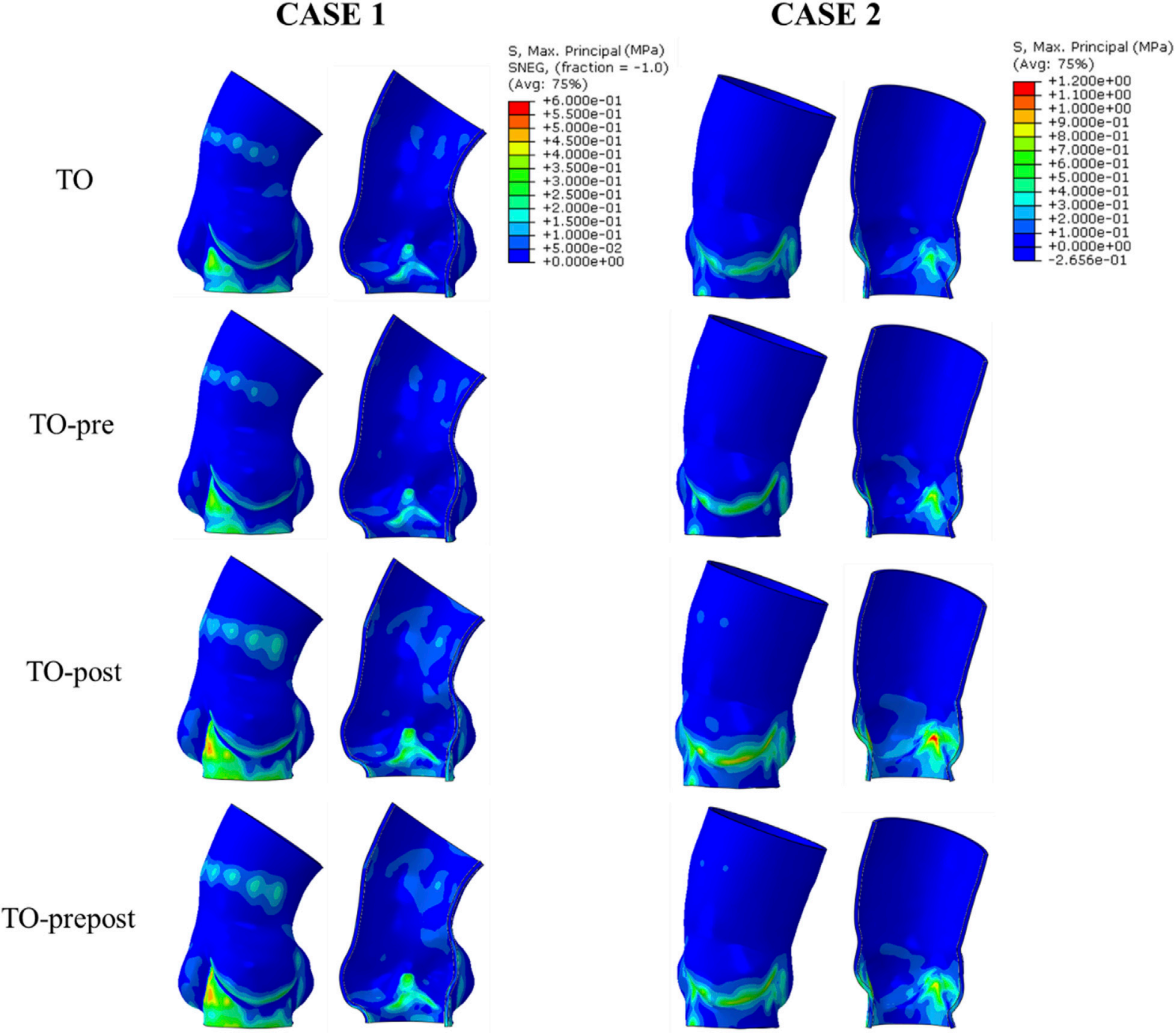
Aortic stress distribution for four balloon dilation models.

Figure 4 shows the stress distribution of the aortic root with different balloon dilation in two cases. In both cases, areas of high stress were found at the inferior edge of the aortic sinuses and in the intra-triangle. The stress at the inferior edge of the sinus increased due to the global dilation of the aortic root caused by the valve stent, while the high stress in the triangular area of the sinus was due to the pull effect in different direction acting on the commissure edge of the native valve when the stent pushes them aside. Balloon dilation made no effect on the location of stress concentration. The peak stress results at the aortic root in the two cases were listed in Table 3. In the two cases, the peak stress of the aorta in the TO and TO-pre models did not vary significantly, indicating that pre-dilation had little impact on the final stress results of the aortic root. Both the TO-post and TO-prepost models that included the post-dilation step showed a significant increase in aortic stress, indicating that post-dilation caused the stent to exert a greater force on the aorta.

**TABLE 3:** Aortic stress in different models for two cases.

	Model	Peak stress (MPa)	Percentage increase compared to TO model
CASE1	TO	0.66	0%
	TO-pre	0.58	−12.2%
	TO-post	0.78	18.2%
	TO-prepost	0.74	12.1%
CASE2	TO	0.76	0%
	TO-pre	1.00	31.6%
	TO-post	1.35	77.7%
	TO-prepost	1.21	59.2%

### 3.2 Deformation of prosthetic stent

**FIGURE 5:**
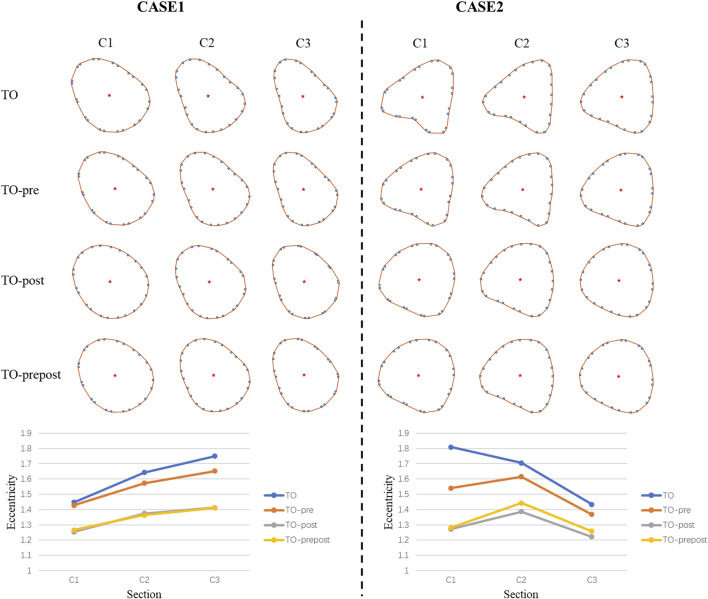
Cross-section shape and eccentricity of stents in each model.

Figure 5 shows the cross-sectional deformation and eccentricity after stent implantation in each model for two cases. The morphology of the stent showed an important relationship with the distribution of calcification. In CASE 1, the cross-section of the stent was constrained by the shape of the native valve to be an elliptical shape. The balloon post-dilation made it to open more fully along the short axis. In CASE 2, because there was also a large amount of calcification on the ventricular side of the native valve, the stent was limited by the calcification mass, and the cross-section was in the shape of a concave arc triangle. The balloon post-dilation effectively reduced the degree of concavity of the stent, allowing it to better restore its original shape. In the two cases, the eccentricity of stent showed almost no difference between TO-post and TO-prepost model, and both of their eccentricity were significantly lower than TO model. However, cross-sectional morphology and eccentricity of the TO-pre model were not significantly improved compared to the TO model.

### 3.3 Analysis of paravalvular leak

**FIGURE 6:**
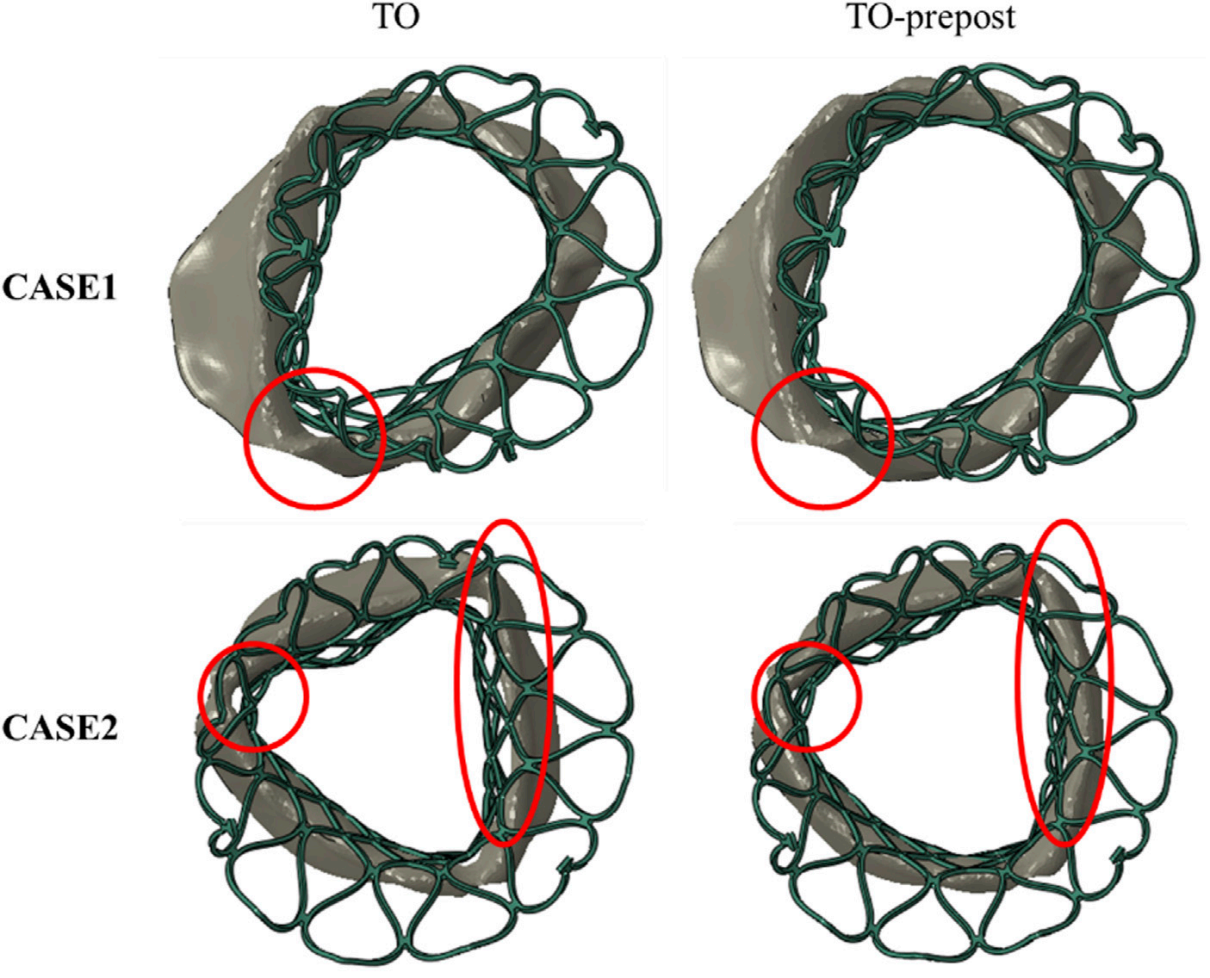
Stent adherence after implantation with/without balloon dilation in two cases.

Since the calcified valve could not fully expand, the stent was blocked by the apposition area of the leaflets during releasing, so that it could not fit well to the wall at the commissure edge, forming a significant gap area, as shown in Figure 6. After balloon dilation, the adherence of the stent in the TO-prepost model was significantly improved, and the gaps at the commissure edge were eliminated or reduced.

**FIGURE 7:**
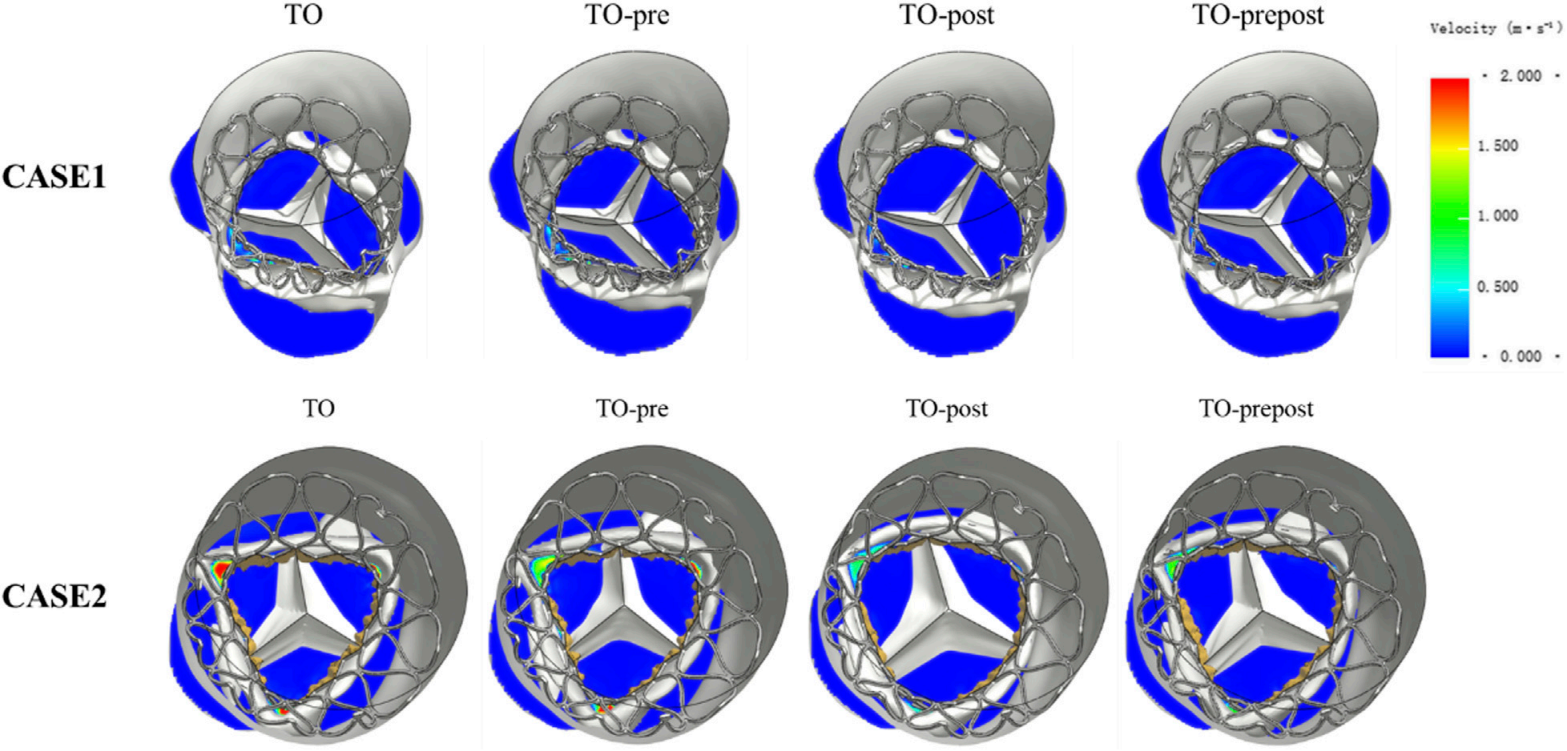
Paravalvular leak patterns for different models.

Figure 7 shows the location of paravalvular leak in each model for the two cases. The occurrence of paravalvular leak was mainly concentrated at the commissure edge of the native valve, which was consistent with the location of the gap formed by stent inadequate apposition. The regurgitant volume could be calculated from flow. Assuming a normal physiological cardiac output of 5.0 L after TAVR, the regurgitant fraction of paravalvular leakage was calculated and the regurgitation grading of paravalvular leakage was obtained according to the clinical criteria (Dvir et al., 2018). Table 4 lists the regurgitation flow in each model. Since the degree of calcification in CASE 2 was more severe than that in CASE 1, and the calcification was distributed on both sides of the valve, the regurgitation volume of each model in CASE 2 was much higher than that of the CASE 1. For CASE 1, the stroke regurgitation volume of the TO-post model decreased by 36.3% and the TO-prepost model decreased by 29.7%, and the degree of regurgitation decreased from “mild” to “moderate to mild”. For CASE 2, the TO-post model’s stroke regurgitation volume decreased by 56.9%, the TO-prepost model decreased by 54.6%. The regurgitation degree decreased from “moderate to severe” to “moderate”. In these two cases, there was almost no change in the regurgitation volume and paravalvular leak degree of the TO-pre model.

**TABLE 4:** Regurgitation degree in different models for two cases.

	Model	Flow (mL/s)	Regurgitant volume (mL)	Regurgitant fraction (%)	Regurgitant grading
CASE1	TO	8.6	4.5	6.7	Mild to moderate
	TO-pre	8.9	4.6	6.9	Mild to moderate
	TO-post	5.5	2.8	4.3	Mild
	TO-prepost	6.0	3.1	4.7	Mild
CASE2	TO	33.7	17.5	26.3	Moderate to severe
	TO-pre	33.8	17.6	26.4	Moderate to severe
	TO-post	14.5	7.6	11.3	Moderate
	TO-prepost	15.3	7.9	11.9	Moderate

## 4 Discussion

This study constructed a complete patient specific aortic model based on CTA imaging, including the aortic root and native valve with calcification. The implantation process of self-expandable valve considering balloon pre-dilation and post-dilation in the pathological model was established, and the effect of two balloon dilation methods was compared. The results showed that balloon dilation has a significant impact on post-operative outcomes.

Both balloon pre-dilation and post-dilatation are part of the surgical procedures for TAVR, but the main purpose of pre-dilation is to open of the native calcified leaflets. The simulation results indicated that pre-dilation only makes a little impact on post-operative aortic stress, stent morphology and paravalvular leak. The balloon post-dilation step is usually used when the implanted valve did not expand enough, so it plays a major role in post-operative outcomes. Balloon post-dilation significantly increase the interaction between the stent and the aortic wall, resulting in increased stress in the aortic root and better valve anchorage. However, stress concentration will also increase the risk of aortic rupture during valve implantation. In addition, higher stress may cause calcification and tissue shedding on aorta, which may block vessels in the brain and lead to stroke. Some studies had also demonstrated the association of shedding tissue with stroke (Nombela-Franco et al., 2012; Van Mieghem et al., 2015). According to the research of Abbasi et al., insufficiently deployed stent will cause increased stress on the prosthetic leaflets, which may reduce the durability of the valve (Abbasi and Azadani, 2015; Nappi et al., 2021). Balloon post-dilation can fully expand the valve stent, effectively reduce the eccentricity of the stent and maintain a better circular cross-section, therefore improve the performance and durability of the valve. The area of post-operative paravalvular leak may be a potential location for long-term thrombosis (Bianchi et al., 2019), and the degree of paravalvular leak is related to the patient’s long-term survival rate (Maisano et al., 2015). Balloon post-dilation improves the adherence of the stent and effectively reduces the degree of paravalvular leak, which is also consistent with clinical findings (Barbanti et al., 2014; Miyasaka et al., 2018).

In summary, the main reason why the balloon post-dilation step can improve TAVR outcomes is that the balloon expansion process makes the valve a better deployed state. The mechanical mechanism behind it is mainly that the balloon fully deforms the calcified native leaflets, which “soften” the whole structure by a larger force. The second explanation is related to the special mechanical properties of loading and unloading of nickel-titanium materials. Although in a same compressed diameter, the nitinol stent acts out different radial force between the loading and unloading process (Tzamtzis et al., 2013; Stoeckel et al., 2019). For the process of simply releasing the stent, the stent experiences the unloading process to recover its deformation and reaches equilibrium with the blood vessel under the action of chronic outward force. However, the balloon post-dilation step allows the stent to be released to a larger diameter first. After the balloon is retracted, due to the recovery of the blood vessel, the stent is subjected to a loading process and will produce a corresponding higher radial resistance force compared to the chronic outward force in releasing process. So the stent can reach a better final expanded state.

Limitations were also existed. Calcification was simplified as linear elastic material. However, balloon dilation often destroys the integrity of calcification actually, thereby helping the valve to expand effectively. Pre-dilation was thought to be helpful in fully stent deployment clinically (Pagnesi et al., 2016; Dumonteil et al., 2019), but the calcification mass could not be destroyed in this simulation study, so it still made a greater impact on the results even with pre-dilation. The analysis of paravalvular leak in this study made an assumption of rigid vessel walls and steady-state flow calculations, ignoring the diameter changes and paravalvular gap changes caused by aorta compliance during a cardiac cycle, which may cause the calculation results to be underestimated. Fluid-structure interaction analysis that fully considers all components can better capture the information of the fluid, which is also the direction of our future work. In addition, this study only analyzed balloon dilation through simulation and lacked sufficient *in vitro* verification or clinical data support. We hope to supplement these necessary work in future.

## 5 Conclusion

This study established a complete simulation process for TAVR based on patient specific model, including balloon pre-dilation and post-dilation, and compared the impact of two balloon dilation on post-operative outcomes. Balloon pre-dilation makes a limited impact on the results, while post-dilation plays a major role in affecting the post-operative outcomes. Balloon post-dilation allows the stent to fully expand and adhere to the wall better, reducing post-operative paravalvular leak, but inevitably leading to a higher vascular stress and increase the risk of injury. Balloon dilation needs to be fully considered in TAVR analysis.

## Data Availability

The original contributions presented in the study are included in the article/Supplementary material, further inquiries can be directed to the corresponding authors.
